# Magnetoelectric Sensor Operating in *d*_15_ Thickness-Shear Mode for High-Frequency Current Detection

**DOI:** 10.3390/s24082396

**Published:** 2024-04-09

**Authors:** Fuchao Li, Jingen Wu, Sujie Liu, Jieqiang Gao, Bomin Lin, Jintao Mo, Jiacheng Qiao, Yiwei Xu, Yongjun Du, Xin He, Yifei Zhou, Lan Zeng, Zhongqiang Hu, Ming Liu

**Affiliations:** 1 State Grid Sichuan Electric Power Company, Chengdu 610041, China; lfc21@mails.tsinghua.edu.cn (F.L.); garfield7741@126.com (S.L.); zhouyf2614@126.com (Y.Z.); zenglanrachel@126.com (L.Z.); 2 Department of Electrical Engineering, Tsinghua University, Beijing 100084, China; 3 State Key Laboratory for Manufacturing Systems Engineering, Electronic Materials Research Laboratory, Key Laboratory of the Ministry of Education, Engineering Research Center of Spin Quantum Sensor Chips, Universities of Shaanxi Province, School of Electronic Science and Engineering, Xi’an Jiaotong University, Xi’an 710049, China; jingen-wu@xjtu.edu.cn (J.W.); jieqiang.h@stu.xjtu.edu.cn (J.G.); linbomin@stu.xjtu.edu.cn (B.L.); mojintao@stu.xjtu.edu.cn (J.M.); qiaojiacheng1998@stu.xjtu.edu.cn (J.Q.); andyxu418@stu.xjtu.edu.cn (Y.X.); ydu2019@stu.xjtu.edu.cn (Y.D.); hexin666@stu.xjtu.edu.cn (X.H.); zhongqianghu@xjtu.edu.cn (Z.H.)

**Keywords:** current sensor, high frequency, thickness-shear mode

## Abstract

For the application of high-frequency current detection in power systems, such as very fast transient current, lightning current, partial discharge pulse current, etc., current sensors with a quick response are indispensable. Here, we propose a high-frequency magnetoelectric current sensor, which consists of a PZT piezoelectric ceramic and Metglas amorphous alloy. The proposed sensor is designed to work under *d*_15_ thickness-shear mode, with the resonant frequency around 1.029 MHz. Furthermore, the proposed sensor is fabricated as a high-frequency magnetoelectric current sensor. A comparative experiment is carried out between the tunnel magnetoresistance sensor and the magnetoelectric sensor, in the aspect of high-frequency current detection up to 3 MHz. Our experimental results demonstrate that the *d*_15_ thickness-shear mode magnetoelectric sensor has great potential for high-frequency current detection in smart grids.

## 1. Introduction

The detection of high-frequency currents is of great significance in many fields, such as industrial manufacturing, power grid monitoring, electric vehicles, etc. For example, a great number of high-frequency transient current signals are generated in smart grids when switch operating, and these signals could be great threats to the secondary equipment. To prevent the damage caused by the high-frequency transient current signals and improve the efficiency and security of power grid operation, it is necessary to effectively detect these signals during the operation of the power grid [1,2,3]. In the field of industrial electronics, high-frequency current sensors can be used to calibrate test signals in terms of the electromagnetic interference (EMI) and electromagnetic compatibility (EMC) of electronic equipment. In the case of electrical circuitry, when a quantity of current is flowing from the protective ground conductor, this is called the leakage current, mostly manifested as transient current. With the widespread use of electronic switch technology in the power supply system and corresponding equipment, high-frequency harmonic voltage and high-frequency harmonic current are produced in the circuit. These high-frequency signals are also harmful to humans when flowing through the body; therefore, the detection of a high-frequency signal is needed in the measurement of leakage current [4,5]. Moreover, lightning current has the characteristics of high frequency and large amplitude. It can inadvertently cause great damage to the industrial equipment. Thus, lightning current presents a great challenge for various electrical equipment, and the accurate measurement of lightning current is very important. To better understand the characteristics of lightning current and improve lightning current protection, the acquisition of actual lightning current parameters is also necessary [6,7].

At present, the current sensors used for high-frequency current detection include shunt sensors, optical current sensors, hall current sensors, current transformers (CTs), Rogowski coils (RCs), magnetoresistive sensors, etc. [8] The principle of a shunt sensor is simple, revealing a great ability to resist electromagnetics, and it has excellent measurement accuracy and bandwidth. However, it is a kind of contact measurement and cannot be used to measure large currents. The optical current sensor theoretically has a dynamic response range, wide bandwidth, and good accuracy [9,10], but ambient changes, including vibration and temperature, can easily make the output signal unstable, resulting in low sensing accuracy. Hall current sensors are widely used because they have low cost and are suitable for the measurement of arbitrary waveform current, but their working frequency bandwidth only covers the frequency range from DC to kilohertz [11,12,13,14] and so cannot be used to measure high-frequency currents (Megahertz); further, their accuracy is susceptible to temperature [15,16,17]. Current transformers (CTs) and Rogowski coils (RCs) are based on the principle of electromagnetic induction; they have wide frequency bandwidth, good measurement accuracy, and measurement stability [18,19,20], but the current transformer cannot be used to measure large currents. In addition, Rogowski coils reveal poor performance in a low-frequency and low-current measurement environment.

Depending on the device structure, the magnetoresistive sensors can be classified into anisotropic magnetoresistive (AMR) sensors, giant magnetoresistance (GMR) sensors, and tunnel magnetoresistance (TMR) sensors [21,22]. Among most magnetoresistive sensors, TMR sensors present the most satisfactory performance. TMR current sensors are based on the tunneling magnetoresistance effect, which features better accuracy than that of AMR sensors and GMR sensors. However, the accuracy of magnetoresistive sensors will decrease sharply under the measurement of high-frequency current (Megahertz). Also, TMR current sensors have a high cost in manufacturing. As a result, there still remains an urgent demand for a lower-cost current sensor that can be used for the measurement of a high-frequency (Megahertz) current.

In this study, we propose a thickness-shear mode magnetoelectric (ME) sensor, which consists of a PZT piezoelectric layer and Metglas amorphous alloy. The proposed sensor is designed to work under *d*_15_ thickness-shear mode, where the PZT piezoelectric ceramic is transversally poled, and the Metglas amorphous alloy operates in length extension vibration mode. Optimized performance is obtained in the measurement of a high-frequency current. Compared with the TMR current sensor, the proposed sensor reveals superior performance in the detection of high-frequency current, demonstrating great potential for high-frequency applications.

## 2. Device Design and Fabrication

**Figure 1:**
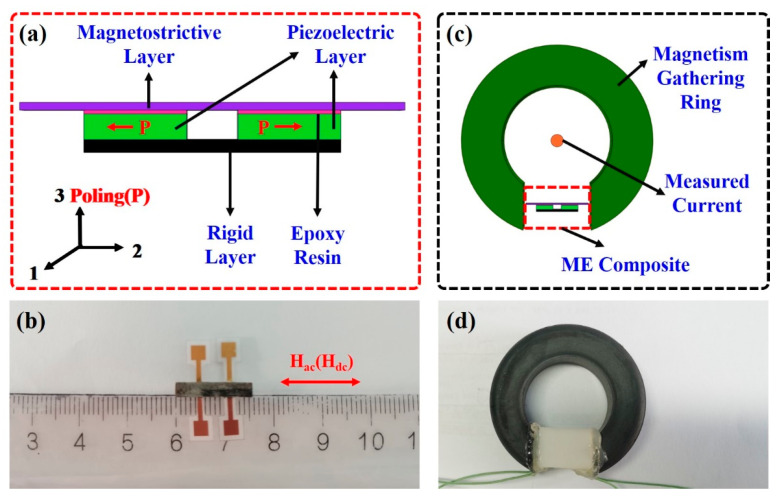
(**a**) Schematic diagram and (**b**) photo of the proposed current sensor. (**c**) Schematic diagram and (**d**) photo of the proposed current sensor installed in magnetism gathering ring.

A schematic diagram of the proposed current sensor is shown in Figure 1a, where the ME composite is composed of a magnetostrictive layer, two piezoelectric layers, and a rigid layer, with two piezoelectric layers sandwiched between the magnetostrictive layer and rigid layer. The two piezoelectric layers are made of PZT piezoelectric ceramic, with dimensions of 4 mm (length), * 2.5 mm (width), * 1 mm (thickness). The two piezoelectric layers are transversally poled the along length direction under an electric field of 1 kV/mm in the silicone oil at 60 °C for 20 min. The poling directions of two piezoelectric layers are set to be the opposite, forming a differential enhancement. Both the top and bottom surfaces of the two piezoelectric layers are covered with conductive epoxy. The magnetostrictive layer is a multilayered structure made of twelve Metglas amorphous alloy layers, bonded together layer by layer. The dimensions of each Metglas amorphous alloy layer are 15 mm (length), * 2.5 mm (width), * 0.02 mm (thickness). The saturation magnetostriction of the Metglas amorphous alloy is around 40 ppm, and the saturation field of the Metglas amorphous alloy is above 100 Oe [23,24]. The rigid layer is made of an acrylic plate, cut to a size of 10 mm (length), * 2.5 mm (width), * 2 mm (thickness). The magnetostrictive layer, rigid layer, and piezoelectric layer are bonded together by epoxy resin cured under room temperature for 24 h. Also, flexible electrodes are connected to both the top and bottom surfaces of the two piezoelectric layers, so as to read the piezoelectric signal. The total length of the proposed current sensor is around 15 mm, with the total thickness below 5 mm (see the photo in Figure 1b). To realize the thickness-shear vibration in the PZT ceramic, the magnetostrictive layer is excited to induce length longitudinal vibration, i.e., the transversal magnetic field (Hac or Hdc) is applied to the ME composite.

The proposed sensor operates in *d*_15_ thickness-shear mode. There are, in total, four working modes in piezoelectric ceramics, i.e., longitudinal (*d*_33_), transversal (*d*_31_), thickness-shear (*d*_15_), and face-shear (*d*_36_). The difference between *d*_15_ thickness-shear mode and other working modes lies in strain type [25]. As for *d*_15_ thickness-shear mode, the output signal of *d*_15_ thickness-shear mode is obtained across the thickness direction, even though it possesses transversal polarization. The piezoelectric ceramic is transversely poled, while the magnetostrictive strain is the longitudinal type. According to this sensor structure, we can derive constitutive equations. The magnetostrictive strain can be calculated as [26]

*σ* = *d*_33,*m*_·*H*_3_, (1)

where *σ* is the magnetostrictive strain, *d*_33,*m*_ is the piezomagnetic coefficient of Metglas (with unit of nm/A), and *H*_3_ is the applied magnetic field.

As for the piezoelectric ceramic, the shear strain can be calculated as [27]

*γ* = *τ*/*G*, (2)

where *γ* is the shear strain, *τ* is the shear stress, and *G* is the shear modulus. Based on the sensor structure, we have σ = 2*γ*. *G* can be calculated as [27]

*G* = *E*/[2(1 + *ν*)], (3)

where *E* is the elasticity modulus of the piezoelectric ceramic, and *ν* is the Poisson ratio of the piezoelectric ceramic. The proposed sensor has differential output. Then, the quasi-static ME coefficient (mV/cm Oe) for the proposed sensor can be calculated as

(4) $$\alpha_{\mathrm{ME},15}=k\cdot d_{33,m}{Eg}_{15}/[2(1+\nu )],$$

where *k* (0 ≤ *k* ≤ 1) is the interface coupling coefficient between the piezoelectric ceramic and Metglas; *g*_15_ is the piezoelectric voltage coefficient of the piezoelectric ceramic. Based on the material parameters [26,28], the quasi-static ME coefficient of the proposed sensors is estimated to be 2.78 mV/cm Oe, which is comparable to other shear-type ME sensors [25].

The designed current sensor adopts non-intrusive current detection; thus, the laminated ME composite is installed in the gap of the magnetism gathering ring, which can aggregate the desired magnetic field from the input current. The magnetism gathering ring is made of MnZn ferrites, which has a relative permeability of around 2322 [29]. The inside diameter of the magnetism gathering ring is 25 mm, the outside diameter is 44.3 mm, and the thickness is 14.3 mm. Our proposed sensor is placed in the gap of the magnetism gathering ring to function as a current sensor. To reduce magnetic leakage, the gap should be as short as possible. The length of the current sensor is 15 mm; thus, a 17 mm gap is cut in the magnetism gathering ring. As schematically shown in Figure 1c, the current in the wire can be measured by the laminated ME composite. A real picture of the proposed magnetoelectric current sensor is presented in Figure 1d, where the ME composite is packaged in plastic housing that is bonded to the magnetism gathering ring.

**Figure 2:**
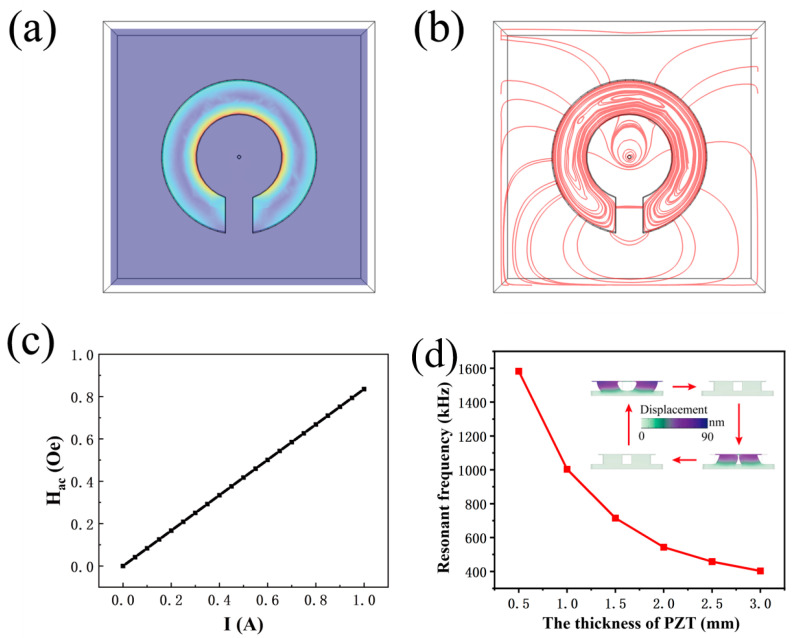
(**a**) The finite element analysis for the magnetic flux density distribution of the proposed magnetism gathering ring. (**b**) The finite element analysis for magnetic line of force. (**c**) The finite element analysis for the linear variation between the power line current and average magnetic field intensity. (**d**) The dependence of resonant frequency with respect to PZT thickness, which was estimated by finite element analysis. The operating mode and deformation displacement of the proposed ME sensor are also indicated.

The output signal amplitude of the sensor can be increased by a suitable design of the magnetism gathering ring. The magnetic field generated by the power line current can converge at the magnetism gathering ring gap to provide a relatively uniform magnetic field for the sensor. Here, we use the finite element analysis (FEA) method to calculate the flux density distribution at the gap of the magnetism gathering ring. The ring’s structure design mentioned in the previous section was imported into COMSOL Multiphysics software 6.0.0.318 (COMSOL Multiphysics software, COMSOL, Inc., Stockholm, Sweden), with its center passing through a power line. The power line is modeled with a radius of 1 mm and wrapped externally with a cubic air domain, with a side length of 100 mm. The material of the magnetism gathering ring was set to be 1J85 Permalloy, which was provided with permeability similar to that of MnZn ferrites, and the power line material was copper. When the 50 Hz AC current flows through the power line, a circular magnetic field centered on the power line is generated. The magnetic flux density distribution of the proposed sensor can be obtained by finite element analysis, which is shown in Figure 2a.

Figure 2b demonstrates the distribution of magnetic force lines obtained via finite element analysis, and it can be seen that the magnetism gathering ring produces a converging effect on the magnetic field; further, the magnetic field distribution at the gap is relatively uniform. The relation between the magnetic field magnitude and the current magnitude can be obtained by taking the center of the magnetism gathering ring gap as the reference point for the magnetic field magnitude calculation, as shown in Figure 2c. It can be seen that the magnetic field magnitude at the gap of the magnetism gathering ring is proportional to the current magnitude, demonstrating that the current in the power line can be directly measured by the sensor installed at the gap of the magnetism gathering ring. The resonant frequency of the proposed sensor reveals a strong dependence on the PZT thickness, and this is confirmed through finite element analysis. As shown in Figure 2d, the resonant frequency dramatically decreases with the increased PZT thickness. The thickness-dependent resonant frequency makes the proposed *d*_15_ thickness-shear mode sensor competitive in realizing the MHz current measurement, because regulable resonant frequency can be obtained by adjusting the PZT thickness. Figure 2d also demonstrates the operating mode of the proposed sensor. Under the transversally applied magnetic field, the magnetostrictive material produces an extend–retract deformation along the length direction, driving the ceramic to produce the shear strain. Thus, the deformation of *d*_15_ thickness-shear mode is revealed through the four states shown in Figure 2d. According to the simulation results, the maximum deformation of the sensor is about 90 nm. *d*_15_ of PZT ceramics is around several hundred pm/V [28], and, thus, *d*_15_ thickness-shear mode induces strain of several hundred pm, which is at least one order of magnitude smaller than the maximum deformation of 90 nm. Thus, 90 nm is enough for to excite *d*_15_ thickness-shear mode.

**Figure 3:**
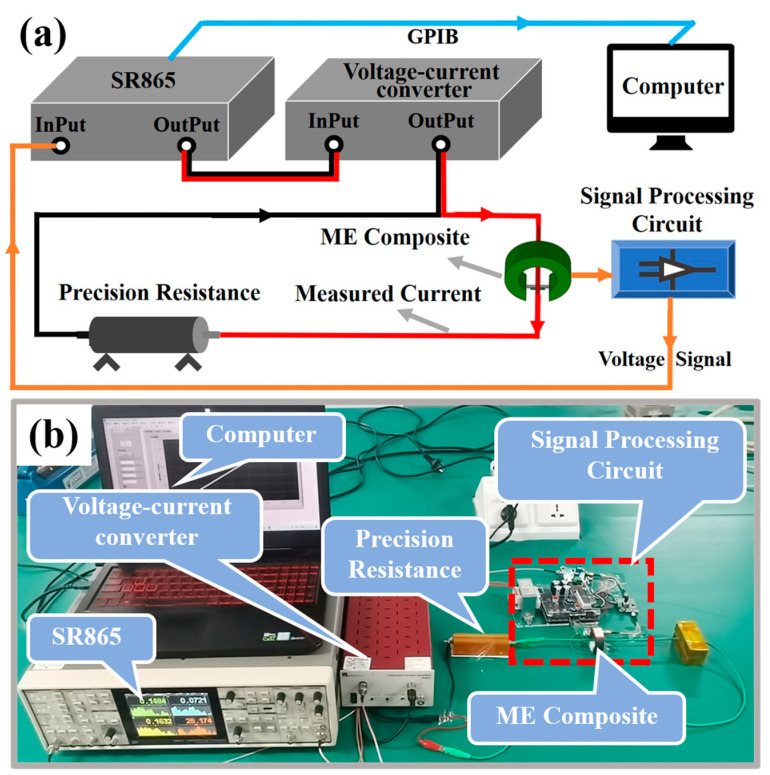
(**a**) Schematic diagram of the test system. (**b**) Photo of the test system.

A schematic diagram and real picture of the test system are shown in Figure 3a,b, respectively. We use LabVIEW software on a PC to control the SR865 lock-in amplifier (LIA) for testing via a GPIB connection. The signal provided by the LIA is loaded onto precision resistance after voltage–current conversion, through the LPA01 voltage–current converter. The wire passes through the center of the magnetism gathering ring, and the sensor is installed in the gap. The output signal of the sensor is captured by the LIA after passing through the signal processing circuit and delivered to LabVIEW software on the PC. With this system, we can test the resonant frequency, bandwidth, limit of detection (LOD), and other technical parameters of the proposed sensor.

## 3. Results and Discussion

**Figure 4:**
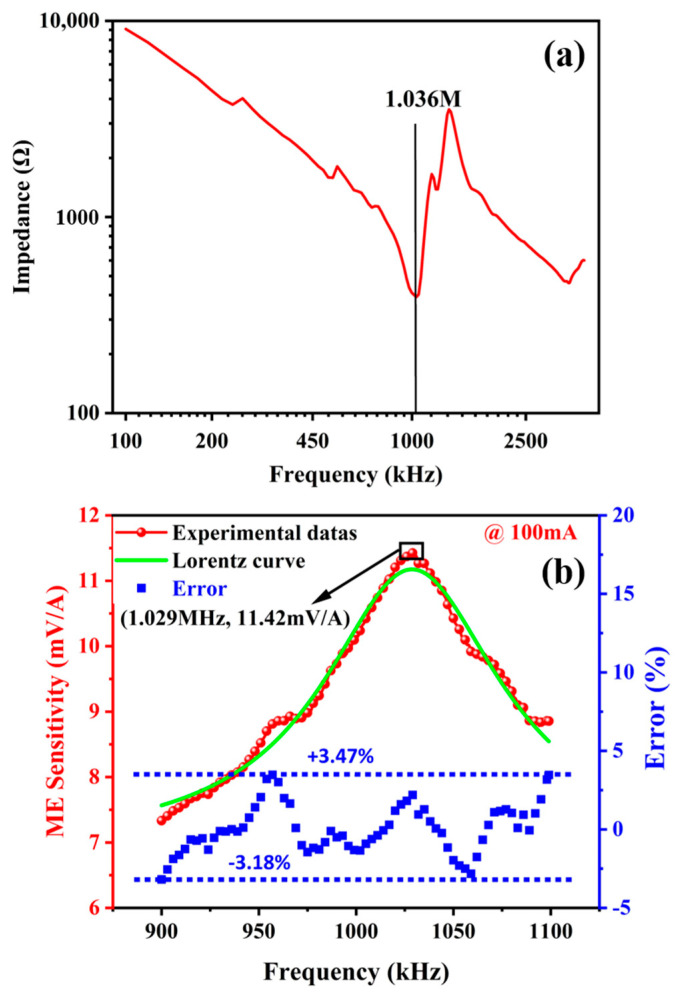
Characterizations for high-frequency resonance performance of the ME composite. (**a**) Impedance spectrum; (**b**) frequency response curve for sensitivity, in 1 MHz ± 10% band range.

The above FEA simulation theoretically demonstrates the mechanical resonance of the proposed sensor. As for the proposed sensor with PZT dimensions of 4 mm (length), * 2.5 mm (width), * 1 mm (thickness), the resonant frequency for *d*_15_ thickness-shear mode is estimated to be around 1.003 MHz, see Figure 2d. In order to confirm the existence of high-frequency electromechanical resonance mode, we measured the impedance spectrum of the ME composite, as shown in Figure 4a. From the results, it can be seen that the resonant peak at 1.036 MHz corresponding to the thickness-shear mode is visibly prominent, coinciding well with the result of FEA simulation. Furthermore, we measured the frequency response curve for the sensitivity of the ME current sensor in the 1 MHz ± 10% frequency band range, as shown in Figure 4b. It can be seen that the proposed ME current sensor, consisting of the laminated ME composite and a magnetism gathering ring, reveals a resonance-enhanced sensitivity up to 11.42 mV/A at 1.029 MHz, which is also consistent with the resonance frequency shown in Figure 4a. It should be noted that both eddy current loss and the Faraday effect are more pronounced when the ferrite magnetism gathering ring is operated in the MHz AC magnetic field, which weakens the ME coupling effect near the high-frequency resonance. Thus, there remains a significant challenge in terms of high-frequency current detection.

**Figure 5:**
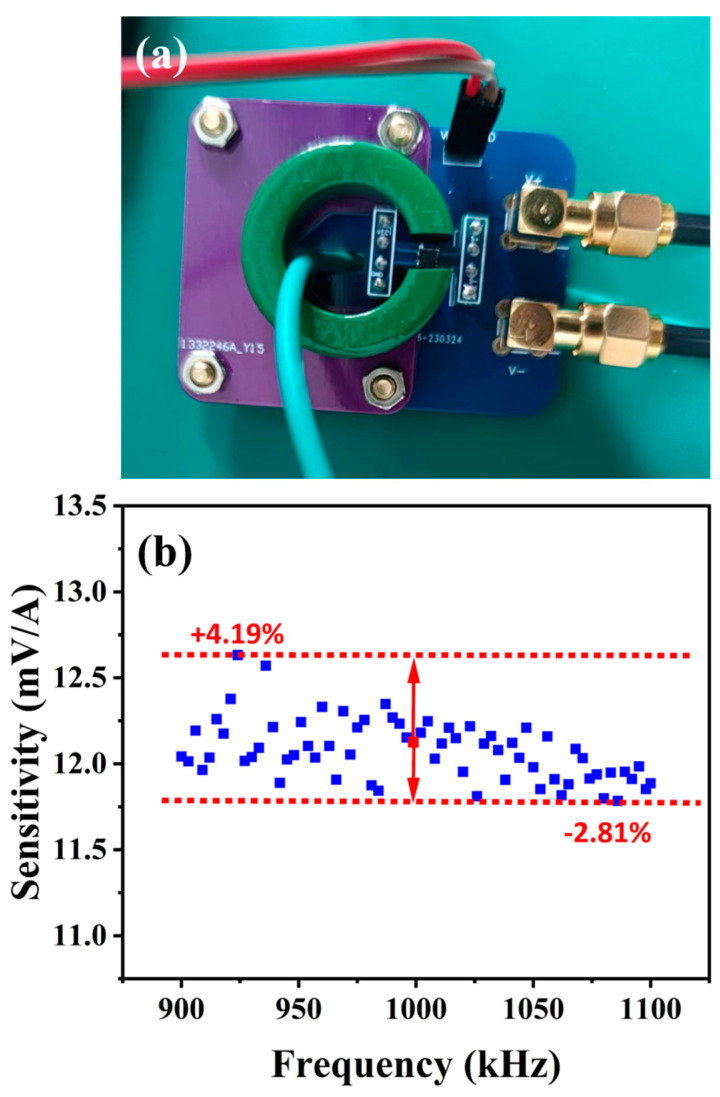
(**a**) Photo of the TMR current sensor. (**b**) Frequency response curve of the TMR current sensor, in 1 MHz ± 10% frequency band range.

To highlight the high-frequency sensing performance of the proposed ME current sensor, we selected a commercial TMR current sensor (Magic2001, Zhuhai MitMagic Technology Co., LTD, High-tech Zone, Zhuhai, China) to perform the comparative experiment, in the 1 MHz ± 10% frequency band range. A picture of the commercial TMR current sensor is presented in Figure 5a. The measured sensitivity curve of the commercial TMR current sensor is shown in Figure 5b. It can be found that the sensitivity of the TMR sensor near 1 MHz reveals some random fluctuations and presents an overall trend of gradual deterioration. The fluctuations (or errors) in the TMR current sensor were calculated by referring to the sensitivity value at 1 MHz, where the fluctuations are in a range of −2.81% to 4.19%.

Differently, the ME current sensor could have a more stable high-frequency performance due to the *d*_15_ thickness-shear vibration mode. Compared with the results of the TMR current sensor, the frequency response curve of the ME current sensor with obvious resonance characteristics can be fitted with a symmetric Lorentz curve, so as to provide a low-error current sensing value read-out. As shown in Figure 4b, the resonance characteristics of the ME sensor were fitted by a symmetric Lorentz curve, and errors were analyzed referring to the Lorentz curve. Based on the Lorentz curve, the frequency dependence characteristics of the ME sensor can be smoothed, and the fluctuations (or errors) in the ME sensor are in a range of −3.18% to 3.47%, which are comparable to those of the TMR current sensor.

**Figure 6:**
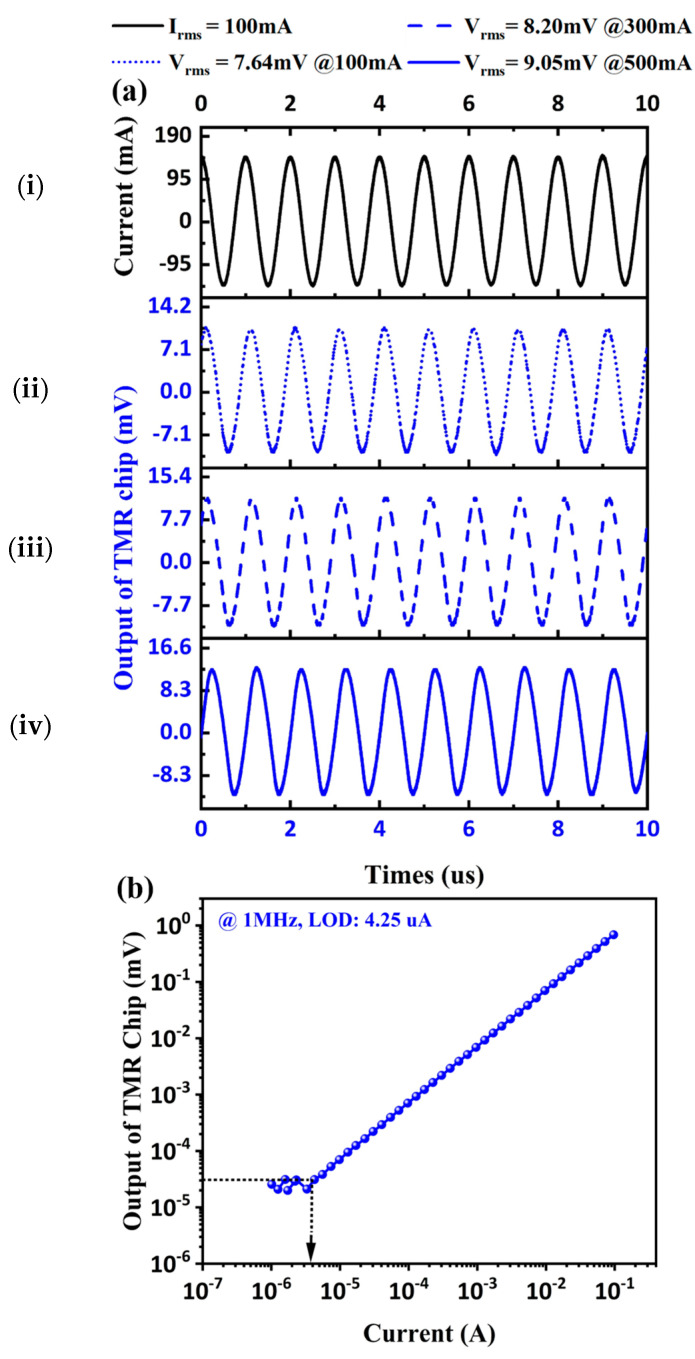
Test results of TMR current sensor for 1 MHz current. (**a**) (**i**) The waveform of input current, output waveforms under the current of different amplitudes: (**ii**) 100 mA, (**iii**) 300 mA, and (**iv**) 500 mA. (**b**) LOD test under 1 MHz.

Further, comparative experiments between TMR and ME current sensors are made, in the aspect of 1 MHz current detection. Figure 6a(ii–iv) show the output waveform of the TMR current sensor, under 1 MHz currents of 100 mA, 300 mA, and 500 mA amplitudes. The waveform of the 1 MHz 100 mA current is also presented in Figure 6a(i). However, the output waveform of the TMR current sensor could still reflect the current waveform when the input current is above 100 mA. However, there is a significant nonlinear relationship between the output amplitude of the TMR current sensor and the current amplitude. This can be attributed to the TMR sensor performance tending to saturation, when the current on the electric cable is above 100 mA. As shown in Figure 6b, we further tested the current LOD of the TMR current sensor. Through numerical analysis, the sensitivity and LOD of the TMR current sensor are 12 mV/A and 4.25 µA, respectively.

**Figure 7:**
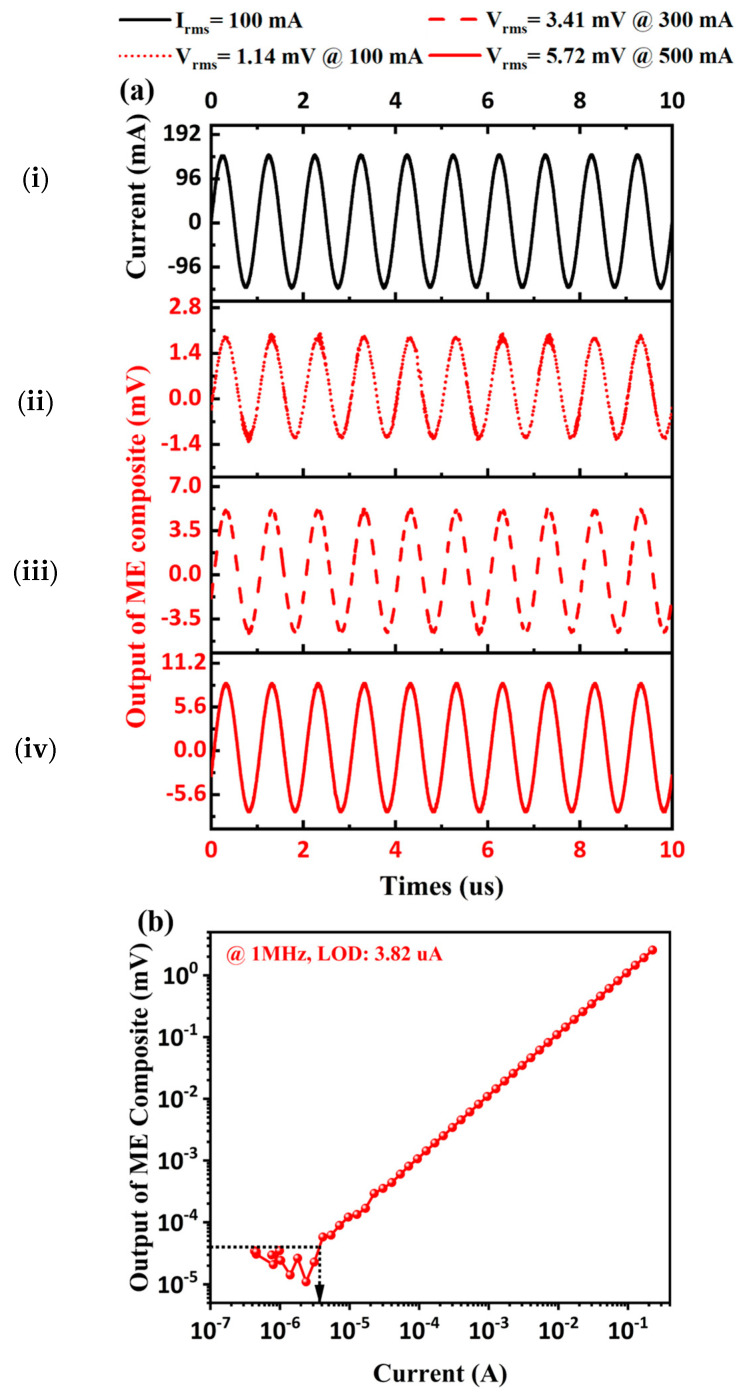
Test results of the proposed ME current sensor for 1 MHz current. (**a**) (**i**) The waveform of input current, output waveforms under the current of different amplitudes: (**ii**) 100 mA, (**iii**) 300 mA, and (**iv**) 500 mA. (**b**) LOD test under 1 MHz.

Similarly, we tested the high-frequency current sensing performance of the proposed ME current sensor under the same conditions. As shown in Figure 7a, when the current applied to the electric cable is above 100 mA, the ME current sensor can still maintain good output linearity and waveform-tracking performance, indicating that it has a larger testing range than that of the TMR current sensor. From the LOD test result in Figure 7b, it can be seen that the proposed ME current sensor has a current sensitivity of about 11.4 mV/A and is able to detect weak current down to 3.82 µA, which can be comparable to the commercial TMR current sensor. The performance of the proposed high-frequency ME current sensor illustrates its application prospect in MHz high-frequency current sensing.

**Figure 8:**
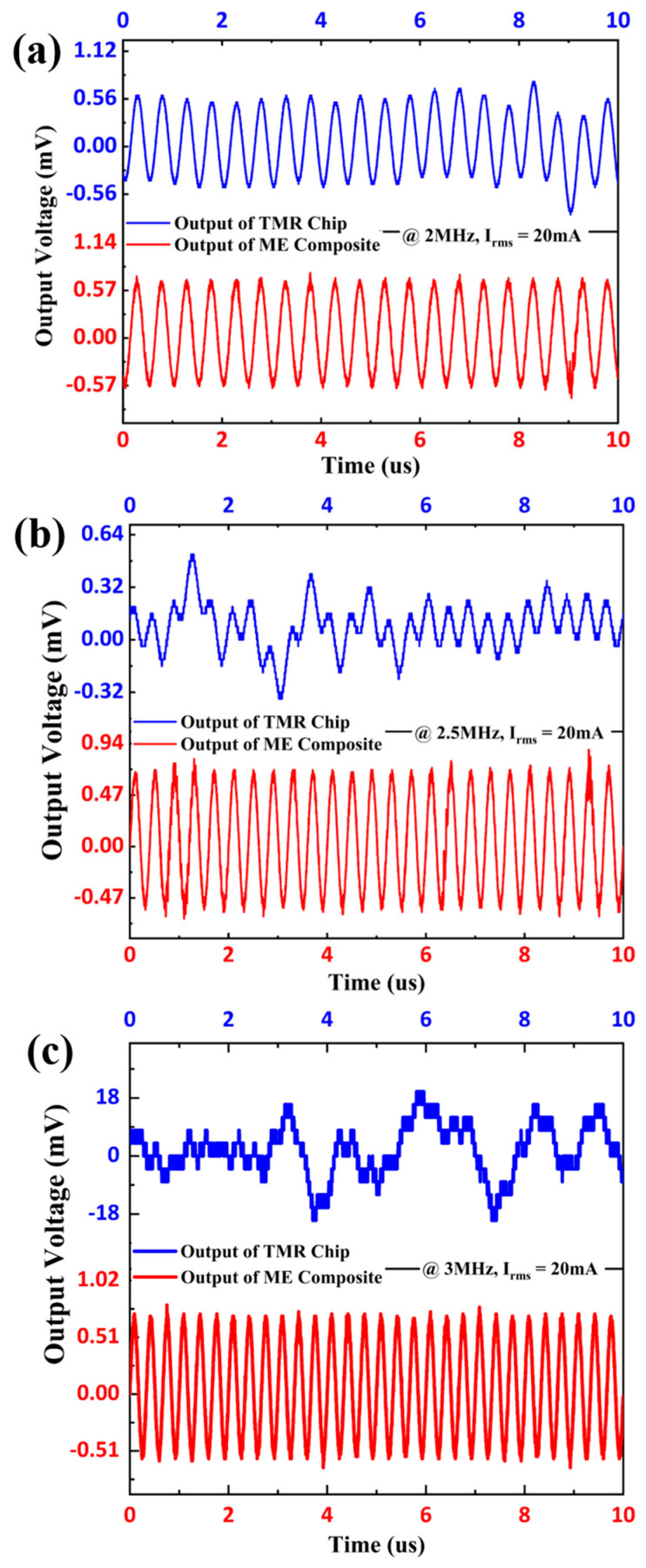
Output voltage waveforms of TMR current sensor and ME current sensor under: (**a**) 2 MHz, (**b**) 2.5 MHz, and (**c**) 3 MHz.

As reported, the typical dynamic frequency ranges for most TMR current sensors were from DC to several hundred kHz or several MHz [30,31,32]. Then, the current test waveforms of TMR and ME current sensors at different high frequencies above 1 MHz were compared, and the results are shown in Figure 8. The high-frequency current detection waveforms of the TMR and ME current sensors at 2 MHz, 2.5 MHz, and 3 MHz frequencies were measured, with the current RMS value fixed at 20 mA. Apparently, the output voltage waveform of the TMR current sensor exhibited serious distortion with the increased frequency, and the output waveform at 3 MHz was completely distorted. Differently, the ME current sensor exhibited a more stable voltage output waveform with increasing frequency up to 3 MHz. The output voltage waveform of the ME current sensor revealed a slight signal burr at the peaks and troughs, which may be due to the high-frequency noise caused by the lack of shielding. Nevertheless, the signal noise of the ME sensor can be reduced by reasonable circuit design [33,34]. Compared to the TMR current sensor, the ME current sensor with a significantly stable voltage output waveform is more capable of high-frequency current detection at the MHz level. This result shows that the proposed high-frequency ME current sensor has an advantage over TMR current sensors for MHz level high-frequency current detection, both in terms of measurement bandwidth and measurement linear range.

In addition, a performance comparison between the proposed ME current sensor and other previously reported ME current sensors is also made. The performance parameters, including measuring range, sensitivity, and working frequency, are listed in Table 1. It can be seen that the proposed ME current sensor presents an acceptable sensitivity, and its measuring range is more than five orders of magnitude higher. Compared with other ME current sensors, the proposed ME current sensor demonstrates potential for wide-range current detection at the MHz frequency level.

## 4. Conclusions

In summary, we proposed a high-frequency magnetoelectric current sensor operating in *d*_15_ thickness-shear mode. The proposed ME current sensor revealed a sensitivity of 11.4 mV/A and current LOD of 3.82 µA under 1 MHz. Furthermore, comparative experiments were made between the proposed magnetoelectric current sensor and the commercial TMR current sensor. The proposed magnetoelectric current sensor revealed similar performance for the commercial TMR current sensor under 1 MHz. Moreover, it was demonstrated that the proposed current sensor presented higher frequency responses up to 3 MHz and larger measurement linear ranges up to 500 mA, compared with the commercial TMR current sensor. Our results prove that magnetoelectric current sensors operating in *d*_15_ thickness-shear mode have great potential for high-frequency current detection at the MHz level.

## Figures and Tables

**Table 1:** Performance comparison between the proposed ME current sensor and other previously reported ME current sensors.

Component	Measuring Range	Sensitivity (mV/A)	Working Frequency	Reference
Terfenol-D/PZT	0.01–2 A	12.6	1 Hz–30 kHz	[35]
Terfenol-D/PZT	0.01–100 A	152	50 Hz	[36]
Metglas/PZT	0.01–5 A	198.91	50 Hz	[37]
Metglas/Ni/PZT	0–2 A	330	50 Hz	[38]
Ni/PZT	0.2–740 A	0.042	50 Hz	[39]
Metglas/PVDF	0–5 A	476.5	28.4 kHz	[40]
Terfenol-D/PZT	0–1000 A	1.014	1 kHz	[41]
NZFO/PZT	0–0.08 A	3.24	1 kHz	[42]
Terfenol-D/PZT	0.015–2.1 A	1.03	50 Hz–5000 Hz	[43]
Metglas/PZT	0–5 A	340	1 kHz	[44]
Metglas/PZT	0–4 A	5426	174.4 kHz	[45]
Terfenol-D/PZT	0–20 A	0.65–12.55	10 Hz–170 kHz	[46]
Metglas/PZT	3.28 μA–0.5 A	7.33~11.4	900 kHz–1100 kHz	This work

## Data Availability

Data are available from the corresponding author upon reasonable request.
